# Supplementary material for: The causal relationship between the human gut microbiota and pyogenic arthritis: a Mendelian randomization study
Source: Front Cell Infect Microbiol. 2024 Nov 26;14:1452480. doi: 10.3389/fcimb.2024.1452480 (PMC11629706; doi:10.3389/fcimb.2024.1452480)
Supplement: Supplementary file 1 [file DataSheet1.zip › mendelian test/ebi-a-GCST90027523.csv_forest.pdf]

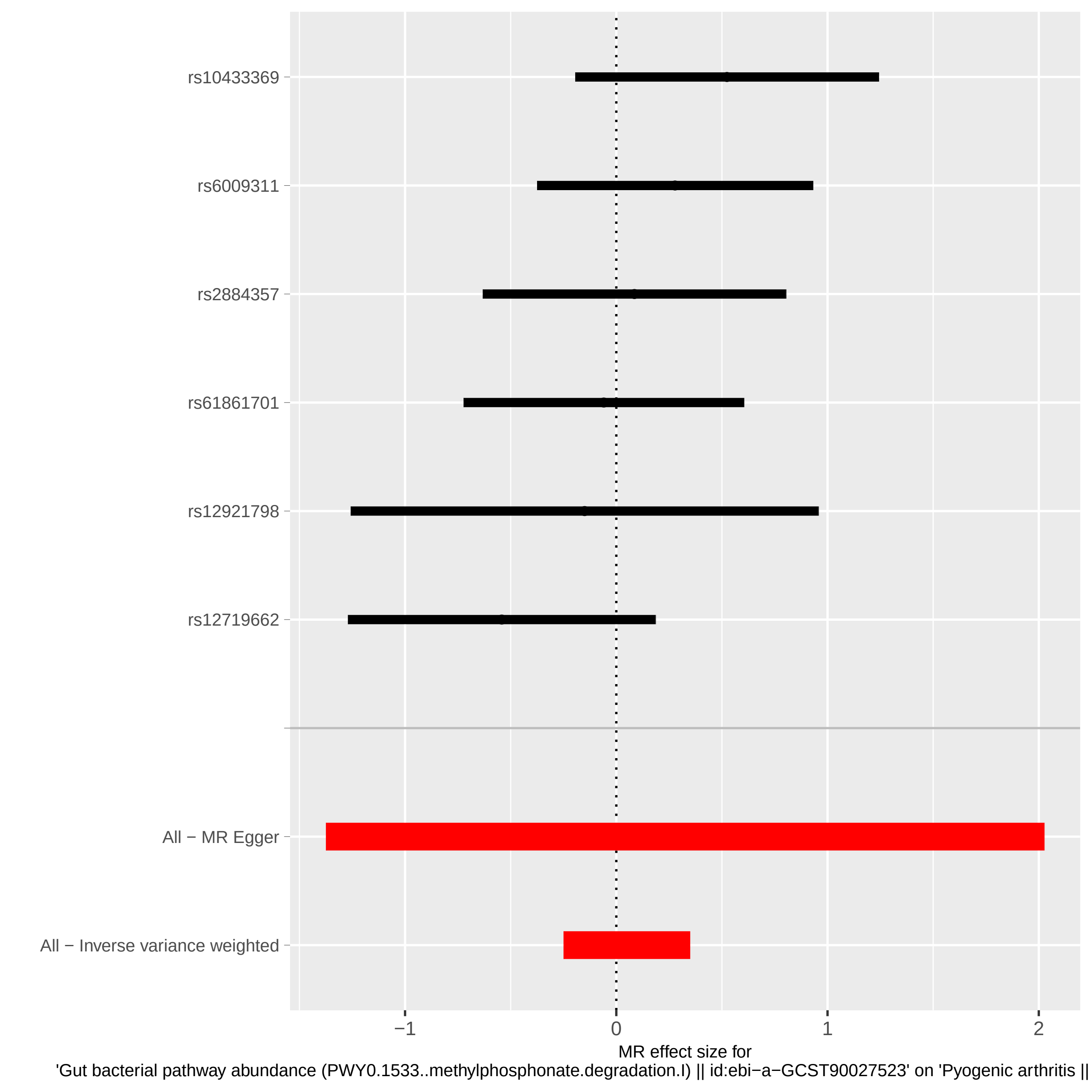

rs10433369
rs6009311
rs2884357
rs61861701
rs12921798
rs12719662
All − MR Egger
All − Inverse variance weighted
−1
0
1
2
MR effect size for
'Gut bacterial pathway abundance (PWY0.1533..methylphosphonate.degradation.I) || id:ebi−a−GCST90027523' on 'Pyogenic arthritis ||
